# The crystal structure of quaternary (Sn,Pb,Bi)Pt

**DOI:** 10.1107/S2056989023000956

**Published:** 2023-02-07

**Authors:** Leonard Rössner, Yurii Prots, Yuri Grin, Marc Armbrüster

**Affiliations:** a Faculty of Natural Sciences, Institute of Chemistry, Materials for Innovative Energy Concepts, Chemnitz University of Technology, 09107 Chemnitz, Germany; b Max-Planck-Institut für Chemische Physik fester Stoffe, Nöthnitzer Strasse 40, 01187 Dresden, Germany; Vienna University of Technology, Austria

**Keywords:** crystal structure, NiAs structure type, single-crystal X-ray diffraction, (Sn,Pb,Bi)Pt

## Abstract

Quaternary (Sn,Pb,Bi)Pt was synthesized by melting of the elements in an evacuated silica glass ampoule. The crystal structure was established by single-crystal X-ray diffraction and adopts an atomic arrangement of the NiAs type with additional occupation of the voids. Decisive for the refinement was the composition of the crystals as determined by energy dispersive X-ray spectroscopy (EDXS), resulting in a formula of (Sn_0.15_Pb_0.54_Bi_0.31_)Pt.

## Abstract 2

The crystal structure of quaternary (Sn,Pb,Bi)Pt adopts the NiAs structure type with additional occupation of voids.

## 1. Chemical context

Platinum-based inter­metallic compounds possess promising properties as electrocatalysts and provide necessary stability for the harsh application conditions in acidic electrolytes (Rössner & Armbrüster, 2019 ▸). SnPt, PbPt and BiPt are inter­esting electrocatalysts for the oxidation of small organic mol­ecules and have the NiAs type of crystal structure (Oftedal, 1928 ▸; Nowotny *et al.*, 1946 ▸; Zhuravlev *et al.*, 1962 ▸). So far, the existence of a substitutional solid solution between PtPb and PtBi was confirmed by powder X-ray diffraction, with the site occupancy deduced from the nominal composition (Zhuravlev *et al.*, 1962 ▸), which also holds for all three binary end members. To obtain material for electrocatalytic investigations, the synthesis of single-phase (Sn,Pb,Bi)Pt was attempted. Large hexa­gonal crystals were found on the top of an otherwise microgranular ingot. Preliminary EDXS analysis indicated the presence of all four elements in the crystal. Further structural investigations besides the original structure reports for PtSn (Harris *et al.*, 1968 ▸; Shelton *et al.*, 1981 ▸; Durussel *et al.*, 1994 ▸), PtPb (Zhuravlev *et al.* 1962 ▸; Sidorov *et al.*, 2021 ▸) and PtBi (Zhuravlev & Stepanova, 1962*a* ▸,*b* ▸) provide no full structural characterization by means of single-crystal X-ray diffraction. Thus, structural data for binary, ternary or quaternary samples in the (Sn,Pb,Bi)Pt system are incomplete. To provide such data, one of the obtained crystals was studied by means of single-crystal X-ray diffraction.

## 2. Structural commentary

**Figure 1:**
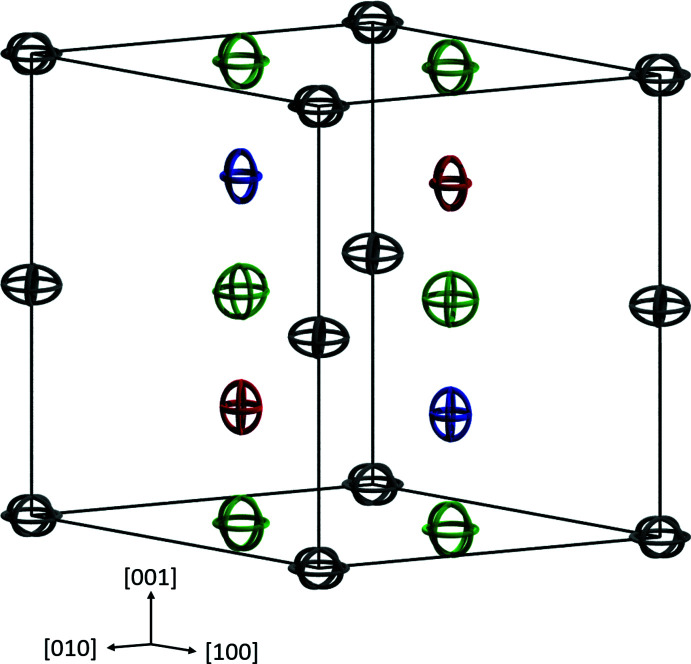
Representation of the unit cell of quaternary (Sn,Pb,Bi)Pt. Color code: Grey – Pt1; red – Sn1, Pb1, Bi1; blue – Sn2; green – Sn3. Displacement ellipsoids are drawn at the 95% probability level..

As a result of the very similar scattering power of three of the four atoms (Bi, Pb and Pt), the direct assignment of the atomic positions to the respective elements was not possible. Atoms were distributed based on crystal-chemical considerations as well as by achieving an agreement between the refined composition and the result of the EDXS analysis (Fig. 1 ▸). The 2*a* site was assigned to Pt in agreement with structural studies of binary endmembers. A mixed occupancy of Sn, Pb and Bi was assumed for the 2*c* position. The statistical distribution of these elements at the same atomic site is based on the full miscibility of the elements in the molten state and on the missing site preference in the only known binary phase Pb_0.7_Bi_0.3_ (Mg type of crystal structure; Kurnakov & Ageeva, 1937 ▸). Additional electron density was detected on the 2*c* ( 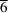 *m*2) and 4*f* (3*m*.) sites, for which two possible scenarios can be considered. Either those positions are occupied by the smaller Sn atoms as a result of the enlarged unit-cell volume of 84.84 Å^3^, which is 7.2% higher compared to 79.14 Å^3^ for SnPt (Oftedal, 1928 ▸), or the presence of stacking faults. Neither can be proven here.

As a result of the potential partial occupation of 2*c* ( 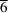 *m*2) and 4*f* (3*m*.) in the hexa­gonal lattice of the quaternary sample, we assign the crystal structure to the NiAs type. The refined composition of 7.5%_at_ Sn, 27.0%_at_ Pb, 15.5%_at_ Bi and 50%_at_ Pt is in broad agreement with the results of EDXS measurements (12.35%_at_ Sn, 25.87%_at_ Pb, 9.49%_at_ Bi and 52.29%_at_ Pt) considering the error of this method, which to our experience is up to 5%_at_ for standardless qu­anti­fications of non-ideal samples, *i.e.* mirror-finished surfaces.

## 3. Synthesis and crystallization

Elements were weighed in an Ar-filled glove-box (O_2_ and H_2_O content < 0.1 ppm) according to the nominal composition of 20.83%_at_ Sn (99.999%, granules, ChemPUR), 20.83%_at_ Pb (99.999%, granules, AlutervFKI), 8.33%_at_ Bi (99.997%, granules, AlfaAesar) and 50.00%_at_ Pt (99.95%, foil, Goodfellow), then sealed in an evacuated silica glass ampoule. The ampoule was placed into a furnace at 1473 K for 24 h, then cooled down from 1473 K to 873 K at a rate of 0.2 K min^−1^. The temperature of 873 K was held for seven days and subsequently the ampoule was quenched in cold water. Single crystals with a hexa­gonal shape were selected from the top of an otherwise microgranular sample, which was composed of phases with the Cu_3_Au and NiAs type of crystal structure, based on powder X-ray diffraction data. As a result of the high X-ray absorption of the investigated material, hexa­gonal-shaped specimens were too large for single crystal X-ray data collection. For this experiment, a relatively small piece was mechanically separated from a hexa­gonally shaped block. The composition of the investigated single crystal was determined by EDXS (Quantax, Bruker).

## 4. Refinement

Crystallographic data, data collection and structure refinement details are summarized in Table 1 ▸.

To decrease the number of parameters, the Pt site was constrained to full occupation at the 2*a* ( 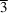 *m*.) site. Even though the standardless qu­anti­fication by means of EDXS data is 52.3%_at_ Pt, recent results of bulk samples from the quasi-ternary cut of the quaternary Sn–Pb–Bi–Pt system indicate a strict upper compositional limit of 50%_at_ Pt (Rössner *et al.*, 2023 ▸). An initial refinement was done for Pb and Bi, using EDXS values as a starting point, then the additional electron density was considered by adding Sn. After multiple cycles, it was decided that a compromise had to be made between excellent refinement results and compositions close to the ones from EDXS results. The final model is presented here.

Furthermore, it has to be noted that Sn3 was refined with isotropic displacement parameters, as the minor site occupancy (2.7%), does not justify to add additional parameters to enable a refinement with anisotropic displacement parameters. It has to be stressed that the ratio of 13 parameters for 123 independent reflections is already at the recommended upper limit (ratio parameters:reflections < 1:10).

## Supplementary Material

Crystal structure: contains datablock(s) I, quarternary. DOI: 10.1107/S2056989023000956/wm5665sup1.cif

Structure factors: contains datablock(s) I. DOI: 10.1107/S2056989023000956/wm5665Isup2.hkl

CCDC reference: 2239531

Additional supporting information:  crystallographic information; 3D view; checkCIF report

## Figures and Tables

**Table 1:** Experimental details

Crystal data	
Chemical formula	(Sn·Pb·Bi)Pt
*M*_r_	389.32
Crystal system, space group	Hexagonal, *P*6_3_/*m* *m* *c*
Temperature (K)	293
*a*, *c* (Å)	4.228 (1), 5.481 (2)
*V* (Å^3^)	84.84 (5)
*Z*	2
Radiation type	Mo *K*α
μ (mm^−1^)	169.4
Crystal size (mm)	0.04 × 0.03 × 0.02

Data collection	
Diffractometer	Rigaku AFC7 four-circle
Absorption correction	Multi-scan (Blessing, 1995 ▸)
*T*_min_, *T*_max_	0.037, 0.081
No. of measured, independent and observed [*I* > 2σ(*I*)] reflections	1549, 123, 120
*R*_int_	0.043
(sin θ/λ)_max_ (Å^−1^)	0.900

Refinement	
*R*[*F* ^2^ > 2σ(*F* ^2^)], *wR*(*F* ^2^), *S*	0.025, 0.041, 1.51
No. of reflections	123
No. of parameters	13
No. of restraints	1
Δρ_max_, Δρ_min_ (e Å^−3^)	2.08, −1.43

Computer programs: *CrystalClear* (Rigaku, 2008 ▸), *SIR-2014* (Burla *et al.*, 2015 ▸), *SHELXL* (Sheldrick, 2015 ▸) and *DIAMOND* (Brandenburg & Putz, 2018 ▸).

## supplementary crystallographic information

### Crystal data

**Table:**

(Sn·Pb·Bi)Pt	*D*_x_ = 15.23 Mg m^−^^3^
*M**_r_* = 389.32	Mo *K*α radiation, λ = 0.710730 Å
Hexagonal, *P*6_3_/*m**m**c*	Cell parameters from 924 reflections
*a* = 4.228 (1) Å	θ = 9.3–43.0°
*c* = 5.481 (2) Å	µ = 169.4 mm^−^^1^
*V* = 84.84 (5) Å^3^	*T* = 293 K
*Z* = 2	Irregular shaped, grey
*F*(000) = 311	0.04 × 0.03 × 0.02 mm

### Data collection

**Table:**

Rigaku AFC7 four-circle diffractometer	123 independent reflections
Radiation source: Sealed Tube	120 reflections with *I* > 2σ(*I*)
Graphite Monochromator monochromator	*R*_int_ = 0.043
Detector resolution: 28.5714 pixels mm^-1^	θ_max_ = 39.8°, θ_min_ = 5.6°
profile data from φ–scans	*h* = −7→5
Absorption correction: multi-scan (Blessing, 1995)	*k* = −6→7
*T*_min_ = 0.037, *T*_max_ = 0.081	*l* = −5→9
1549 measured reflections	

### Refinement

**Table:**

Refinement on *F*^2^	13 parameters
Least-squares matrix: full	1 restraint
*R*[*F*^2^ > 2σ(*F*^2^)] = 0.025	*w* = 1/[σ^2^(*F*_o_^2^) + (0.012*P*)^2^ + 0.2655*P*] where *P* = (*F*_o_^2^ + 2*F*_c_^2^)/3
*wR*(*F*^2^) = 0.041	(Δ/σ)_max_ = 0.013
*S* = 1.51	Δρ_max_ = 2.08 e Å^−^^3^
123 reflections	Δρ_min_ = −1.43 e Å^−^^3^

### Special details

**Table:**

Geometry. All esds (except the esd in the dihedral angle between two l.s. planes) are estimated using the full covariance matrix. The cell esds are taken into account individually in the estimation of esds in distances, angles and torsion angles; correlations between esds in cell parameters are only used when they are defined by crystal symmetry. An approximate (isotropic) treatment of cell esds is used for estimating esds involving l.s. planes.

### Fractional atomic coordinates and isotropic or equivalent isotropic displacement parameters (Å^2^)

**Table:**

	*x*	*y*	*z*	*U*_iso_*/*U*_eq_	Occ. (<1)
Pt1	0	0	0	0.0110 (2)	
Bi1	0.3333	0.6667	0.2500	0.0077 (3)	0.31 (5)
Pb1	0.3333	0.6667	0.2500	0.0077	0.54 (5)
Sn1	0.3333	0.6667	0.2500	0.0077	0.03 (4)
Sn2	0.3333	0.6667	0.7500	0.0077	0.064 (6)
Sn3	0.3333	0.6667	0.501 (5)	0.012 (7)*	0.027 (4)

### Atomic displacement parameters (Å^2^)

**Table:**

	*U*^11^	*U*^22^	*U*^33^	*U*^12^	*U*^13^	*U*^23^
Pt1	0.0133 (3)	0.0133	0.0063 (3)	0.00667 (13)	0	0
Bi1	0.0061 (3)	0.0061	0.0108 (4)	0.00307 (14)	0	0
Pb1	0.0061	0.0061	0.0108	0.00307	0	0
Sn1	0.0061	0.0061	0.0108	0.00307	0	0
Sn2	0.0061	0.0061	0.0108	0.00307	0	0

### Geometric parameters (Å, º)

**Table:**

Pt1—Sn3^i^	2.4410 (6)	Pb1—Sn3^x^	2.797 (13)
Pt1—Sn3^ii^	2.4410 (6)	Pb1—Sn3^xiii^	2.797 (13)
Pt1—Sn3^iii^	2.4410 (6)	Sn1—Sn3	1.37 (3)
Pt1—Sn3^iv^	2.4410 (6)	Sn1—Sn3^i^	1.37 (3)
Pt1—Sn3^v^	2.4411 (6)	Sn1—Sn2^ix^	2.4410 (6)
Pt1—Sn3^vi^	2.4411 (6)	Sn1—Sn2^x^	2.4410 (6)
Pt1—Pt1^vii^	2.7402 (9)	Sn1—Sn2^xi^	2.4410 (6)
Pt1—Pt1^ii^	2.7402 (9)	Sn1—Sn2	2.7403 (8)
Pt1—Bi1^viii^	2.7993 (5)	Sn1—Sn2^xii^	2.7402 (9)
Pt1—Sn1^viii^	2.7993 (5)	Sn1—Sn3^iv^	2.797 (13)
Pt1—Pb1^viii^	2.7993 (5)	Sn1—Sn3^ix^	2.797 (13)
Pt1—Sn2^ix^	2.7993 (5)	Sn1—Sn3^vi^	2.797 (13)
Bi1—Sn3	1.37 (3)	Sn1—Sn3^x^	2.797 (13)
Bi1—Sn3^i^	1.37 (3)	Sn1—Sn3^xiii^	2.797 (13)
Bi1—Sn2^ix^	2.4410 (6)	Sn2—Sn3^xiv^	1.37 (3)
Bi1—Sn2^x^	2.4410 (6)	Sn2—Sn3	1.37 (3)
Bi1—Sn2^xi^	2.4410 (6)	Sn2—Sn1^ix^	2.4410 (6)
Bi1—Sn2	2.7403 (8)	Sn2—Pb1^ix^	2.4410 (6)
Bi1—Sn2^xii^	2.7402 (9)	Sn2—Bi1^ix^	2.4410 (6)
Bi1—Sn3^iv^	2.797 (13)	Sn2—Sn1^x^	2.4410 (6)
Bi1—Sn3^ix^	2.797 (13)	Sn2—Sn1^xi^	2.4410 (6)
Bi1—Sn3^vi^	2.797 (13)	Sn2—Pb1^x^	2.4410 (6)
Bi1—Sn3^x^	2.797 (13)	Sn2—Pb1^xi^	2.4410 (6)
Bi1—Sn3^xiii^	2.797 (13)	Sn2—Bi1^x^	2.4410 (6)
Pb1—Sn3	1.37 (3)	Sn2—Bi1^xi^	2.4410 (6)
Pb1—Sn3^i^	1.37 (3)	Sn3—Sn3^ix^	2.4410 (6)
Pb1—Sn2^ix^	2.4410 (6)	Sn3—Pt1^xv^	2.4410 (6)
Pb1—Sn2^x^	2.4410 (6)	Sn3—Pt1^xvi^	2.4410 (6)
Pb1—Sn2^xi^	2.4410 (6)	Sn3—Pt1^vii^	2.4410 (6)
Pb1—Sn2	2.7403 (8)	Sn3—Sn3^x^	2.4411 (6)
Pb1—Sn2^xii^	2.7402 (9)	Sn3—Sn3^xi^	2.4411 (7)
Pb1—Sn3^iv^	2.797 (13)	Sn3—Sn3^xiv^	2.73 (5)
Pb1—Sn3^ix^	2.797 (13)	Sn3—Sn3^i^	2.75 (5)
Pb1—Sn3^vi^	2.797 (13)		
			
Sn3^i^—Pt1—Sn3^ii^	180.0	Sn3^i^—Sn1—Sn2^x^	90.000 (4)
Sn3^iii^—Pt1—Sn3^iv^	180.0	Sn2^ix^—Sn1—Sn2^x^	120.0
Sn3^i^—Pt1—Sn3^v^	119.999 (5)	Sn3—Sn1—Sn2^xi^	90.000 (5)
Sn3^iii^—Pt1—Sn3^v^	119.999 (4)	Sn3^i^—Sn1—Sn2^xi^	90.000 (4)
Sn3^ii^—Pt1—Sn3^vi^	119.999 (5)	Sn2^ix^—Sn1—Sn2^xi^	120.0
Sn3^iv^—Pt1—Sn3^vi^	119.999 (4)	Sn2^x^—Sn1—Sn2^xi^	120.0
Sn3^v^—Pt1—Sn3^vi^	180.0	Sn3—Sn1—Sn2	0.000 (6)
Sn3^i^—Pt1—Pt1^vii^	90.1 (6)	Sn3^i^—Sn1—Sn2	180.0
Sn3^ii^—Pt1—Pt1^vii^	89.9 (6)	Sn2^ix^—Sn1—Sn2	90.0
Sn3^iii^—Pt1—Pt1^vii^	90.1 (6)	Sn2^x^—Sn1—Sn2	90.0
Sn3^iv^—Pt1—Pt1^vii^	89.9 (6)	Sn2^xi^—Sn1—Sn2	90.0
Sn3^v^—Pt1—Pt1^vii^	90.1 (6)	Sn3—Sn1—Sn2^xii^	180.0
Sn3^vi^—Pt1—Pt1^vii^	89.9 (6)	Sn3^i^—Sn1—Sn2^xii^	0.000 (1)
Sn3^i^—Pt1—Pt1^ii^	89.9 (6)	Sn2^ix^—Sn1—Sn2^xii^	90.0
Sn3^ii^—Pt1—Pt1^ii^	90.1 (6)	Sn2^x^—Sn1—Sn2^xii^	90.0
Sn3^iii^—Pt1—Pt1^ii^	89.9 (6)	Sn2^xi^—Sn1—Sn2^xii^	90.0
Sn3^iv^—Pt1—Pt1^ii^	90.1 (6)	Sn2—Sn1—Sn2^xii^	180.0
Sn3^v^—Pt1—Pt1^ii^	89.9 (6)	Sn3—Sn1—Sn3^iv^	119.2 (5)
Sn3^vi^—Pt1—Pt1^ii^	90.1 (6)	Sn3^i^—Sn1—Sn3^iv^	60.8 (5)
Pt1^vii^—Pt1—Pt1^ii^	180.0	Sn2^ix^—Sn1—Sn3^iv^	29.2 (5)
Sn3^i^—Pt1—Bi1^viii^	150.6 (6)	Sn2^x^—Sn1—Sn3^iv^	115.87 (13)
Sn3^ii^—Pt1—Bi1^viii^	29.4 (6)	Sn2^xi^—Sn1—Sn3^iv^	115.87 (13)
Sn3^iii^—Pt1—Bi1^viii^	64.1 (3)	Sn2—Sn1—Sn3^iv^	119.2 (5)
Sn3^iv^—Pt1—Bi1^viii^	115.9 (3)	Sn2^xii^—Sn1—Sn3^iv^	60.8 (5)
Sn3^v^—Pt1—Bi1^viii^	64.1 (3)	Sn3—Sn1—Sn3^ix^	60.8 (5)
Sn3^vi^—Pt1—Bi1^viii^	115.9 (3)	Sn3^i^—Sn1—Sn3^ix^	119.2 (5)
Pt1^vii^—Pt1—Bi1^viii^	119.305 (9)	Sn2^ix^—Sn1—Sn3^ix^	29.2 (5)
Pt1^ii^—Pt1—Bi1^viii^	60.695 (9)	Sn2^x^—Sn1—Sn3^ix^	115.87 (13)
Sn3^i^—Pt1—Sn1^viii^	150.6 (6)	Sn2^xi^—Sn1—Sn3^ix^	115.87 (13)
Sn3^ii^—Pt1—Sn1^viii^	29.4 (6)	Sn2—Sn1—Sn3^ix^	60.8 (5)
Sn3^iii^—Pt1—Sn1^viii^	64.1 (3)	Sn2^xii^—Sn1—Sn3^ix^	119.2 (5)
Sn3^iv^—Pt1—Sn1^viii^	115.9 (3)	Sn3^iv^—Sn1—Sn3^ix^	58.5 (10)
Sn3^v^—Pt1—Sn1^viii^	64.1 (3)	Sn3—Sn1—Sn3^vi^	119.2 (5)
Sn3^vi^—Pt1—Sn1^viii^	115.9 (3)	Sn3^i^—Sn1—Sn3^vi^	60.8 (5)
Pt1^vii^—Pt1—Sn1^viii^	119.305 (9)	Sn2^ix^—Sn1—Sn3^vi^	115.87 (13)
Pt1^ii^—Pt1—Sn1^viii^	60.695 (9)	Sn2^x^—Sn1—Sn3^vi^	115.87 (13)
Bi1^viii^—Pt1—Sn1^viii^	0.0	Sn2^xi^—Sn1—Sn3^vi^	29.2 (5)
Sn3^i^—Pt1—Pb1^viii^	150.6 (6)	Sn2—Sn1—Sn3^vi^	119.2 (5)
Sn3^ii^—Pt1—Pb1^viii^	29.4 (6)	Sn2^xii^—Sn1—Sn3^vi^	60.8 (5)
Sn3^iii^—Pt1—Pb1^viii^	64.1 (3)	Sn3^iv^—Sn1—Sn3^vi^	98.2 (6)
Sn3^iv^—Pt1—Pb1^viii^	115.9 (3)	Sn3^ix^—Sn1—Sn3^vi^	128.3 (3)
Sn3^v^—Pt1—Pb1^viii^	64.1 (3)	Sn3—Sn1—Sn3^x^	60.8 (5)
Sn3^vi^—Pt1—Pb1^viii^	115.9 (3)	Sn3^i^—Sn1—Sn3^x^	119.2 (5)
Pt1^vii^—Pt1—Pb1^viii^	119.305 (9)	Sn2^ix^—Sn1—Sn3^x^	115.87 (13)
Pt1^ii^—Pt1—Pb1^viii^	60.695 (9)	Sn2^x^—Sn1—Sn3^x^	29.2 (5)
Bi1^viii^—Pt1—Pb1^viii^	0.0	Sn2^xi^—Sn1—Sn3^x^	115.87 (13)
Sn1^viii^—Pt1—Pb1^viii^	0.0	Sn2—Sn1—Sn3^x^	60.8 (5)
Sn3^i^—Pt1—Sn2^ix^	64.2 (3)	Sn2^xii^—Sn1—Sn3^x^	119.2 (5)
Sn3^ii^—Pt1—Sn2^ix^	115.8 (3)	Sn3^iv^—Sn1—Sn3^x^	128.3 (3)
Sn3^iii^—Pt1—Sn2^ix^	150.8 (6)	Sn3^ix^—Sn1—Sn3^x^	98.2 (6)
Sn3^iv^—Pt1—Sn2^ix^	29.2 (6)	Sn3^vi^—Sn1—Sn3^x^	128.3 (3)
Sn3^v^—Pt1—Sn2^ix^	64.2 (3)	Sn3—Sn1—Sn3^xiii^	119.2 (5)
Sn3^vi^—Pt1—Sn2^ix^	115.8 (3)	Sn3^i^—Sn1—Sn3^xiii^	60.8 (5)
Pt1^vii^—Pt1—Sn2^ix^	60.695 (9)	Sn2^ix^—Sn1—Sn3^xiii^	115.87 (13)
Pt1^ii^—Pt1—Sn2^ix^	119.305 (10)	Sn2^x^—Sn1—Sn3^xiii^	29.2 (5)
Sn3—Bi1—Sn3^i^	180.0	Sn2^xi^—Sn1—Sn3^xiii^	115.87 (13)
Sn3—Bi1—Sn2^ix^	90.000 (1)	Sn2—Sn1—Sn3^xiii^	119.2 (5)
Sn3^i^—Bi1—Sn2^ix^	89.999 (1)	Sn2^xii^—Sn1—Sn3^xiii^	60.8 (5)
Sn3—Bi1—Sn2^x^	90.000 (4)	Sn3^iv^—Sn1—Sn3^xiii^	98.2 (6)
Sn3^i^—Bi1—Sn2^x^	90.000 (4)	Sn3^ix^—Sn1—Sn3^xiii^	128.3 (3)
Sn2^ix^—Bi1—Sn2^x^	120.0	Sn3^vi^—Sn1—Sn3^xiii^	98.2 (6)
Sn3—Bi1—Sn2^xi^	90.000 (5)	Sn3^x^—Sn1—Sn3^xiii^	58.5 (10)
Sn3^i^—Bi1—Sn2^xi^	90.000 (4)	Sn3^xiv^—Sn2—Sn3	180.0
Sn2^ix^—Bi1—Sn2^xi^	120.0	Sn3^xiv^—Sn2—Sn1^ix^	89.999 (1)
Sn2^x^—Bi1—Sn2^xi^	120.0	Sn3—Sn2—Sn1^ix^	90.000 (1)
Sn3—Bi1—Sn2	0.000 (6)	Sn3^xiv^—Sn2—Pb1^ix^	89.999 (1)
Sn3^i^—Bi1—Sn2	180.0	Sn3—Sn2—Pb1^ix^	90.000 (1)
Sn2^ix^—Bi1—Sn2	90.0	Sn1^ix^—Sn2—Pb1^ix^	0.0
Sn2^x^—Bi1—Sn2	90.0	Sn3^xiv^—Sn2—Bi1^ix^	89.999 (1)
Sn2^xi^—Bi1—Sn2	90.0	Sn3—Sn2—Bi1^ix^	90.000 (1)
Sn3—Bi1—Sn2^xii^	180.0	Sn1^ix^—Sn2—Bi1^ix^	0.0
Sn3^i^—Bi1—Sn2^xii^	0.000 (1)	Pb1^ix^—Sn2—Bi1^ix^	0.0
Sn2^ix^—Bi1—Sn2^xii^	90.0	Sn3^xiv^—Sn2—Sn1^x^	90.000 (6)
Sn2^x^—Bi1—Sn2^xii^	90.0	Sn3—Sn2—Sn1^x^	90.000 (6)
Sn2^xi^—Bi1—Sn2^xii^	90.0	Sn1^ix^—Sn2—Sn1^x^	120.0
Sn2—Bi1—Sn2^xii^	180.0	Pb1^ix^—Sn2—Sn1^x^	120.0
Sn3—Bi1—Sn3^iv^	119.2 (5)	Bi1^ix^—Sn2—Sn1^x^	120.0
Sn3^i^—Bi1—Sn3^iv^	60.8 (5)	Sn3^xiv^—Sn2—Sn1^xi^	90.000 (7)
Sn2^ix^—Bi1—Sn3^iv^	29.2 (5)	Sn3—Sn2—Sn1^xi^	90.000 (7)
Sn2^x^—Bi1—Sn3^iv^	115.87 (13)	Sn1^ix^—Sn2—Sn1^xi^	120.0
Sn2^xi^—Bi1—Sn3^iv^	115.87 (13)	Pb1^ix^—Sn2—Sn1^xi^	120.0
Sn2—Bi1—Sn3^iv^	119.2 (5)	Bi1^ix^—Sn2—Sn1^xi^	120.0
Sn2^xii^—Bi1—Sn3^iv^	60.8 (5)	Sn1^x^—Sn2—Sn1^xi^	120.0
Sn3—Bi1—Sn3^ix^	60.8 (5)	Sn3^xiv^—Sn2—Pb1^x^	90.000 (6)
Sn3^i^—Bi1—Sn3^ix^	119.2 (5)	Sn3—Sn2—Pb1^x^	90.000 (6)
Sn2^ix^—Bi1—Sn3^ix^	29.2 (5)	Sn1^ix^—Sn2—Pb1^x^	120.0
Sn2^x^—Bi1—Sn3^ix^	115.87 (13)	Pb1^ix^—Sn2—Pb1^x^	120.0
Sn2^xi^—Bi1—Sn3^ix^	115.87 (13)	Bi1^ix^—Sn2—Pb1^x^	120.0
Sn2—Bi1—Sn3^ix^	60.8 (5)	Sn1^x^—Sn2—Pb1^x^	0.0
Sn2^xii^—Bi1—Sn3^ix^	119.2 (5)	Sn1^xi^—Sn2—Pb1^x^	120.0
Sn3^iv^—Bi1—Sn3^ix^	58.5 (10)	Sn3^xiv^—Sn2—Pb1^xi^	90.000 (7)
Sn3—Bi1—Sn3^vi^	119.2 (5)	Sn3—Sn2—Pb1^xi^	90.000 (7)
Sn3^i^—Bi1—Sn3^vi^	60.8 (5)	Sn1^ix^—Sn2—Pb1^xi^	120.0
Sn2^ix^—Bi1—Sn3^vi^	115.87 (13)	Pb1^ix^—Sn2—Pb1^xi^	120.0
Sn2^x^—Bi1—Sn3^vi^	115.87 (13)	Bi1^ix^—Sn2—Pb1^xi^	120.0
Sn2^xi^—Bi1—Sn3^vi^	29.2 (5)	Sn1^x^—Sn2—Pb1^xi^	120.0
Sn2—Bi1—Sn3^vi^	119.2 (5)	Sn1^xi^—Sn2—Pb1^xi^	0.0
Sn2^xii^—Bi1—Sn3^vi^	60.8 (5)	Pb1^x^—Sn2—Pb1^xi^	120.0
Sn3^iv^—Bi1—Sn3^vi^	98.2 (6)	Sn3^xiv^—Sn2—Bi1^x^	90.000 (6)
Sn3^ix^—Bi1—Sn3^vi^	128.3 (3)	Sn3—Sn2—Bi1^x^	90.000 (6)
Sn3—Bi1—Sn3^x^	60.8 (5)	Sn1^ix^—Sn2—Bi1^x^	120.0
Sn3^i^—Bi1—Sn3^x^	119.2 (5)	Pb1^ix^—Sn2—Bi1^x^	120.0
Sn2^ix^—Bi1—Sn3^x^	115.87 (13)	Bi1^ix^—Sn2—Bi1^x^	120.0
Sn2^x^—Bi1—Sn3^x^	29.2 (5)	Sn1^x^—Sn2—Bi1^x^	0.0
Sn2^xi^—Bi1—Sn3^x^	115.87 (13)	Sn1^xi^—Sn2—Bi1^x^	120.0
Sn2—Bi1—Sn3^x^	60.8 (5)	Pb1^x^—Sn2—Bi1^x^	0.0
Sn2^xii^—Bi1—Sn3^x^	119.2 (5)	Pb1^xi^—Sn2—Bi1^x^	120.0
Sn3^iv^—Bi1—Sn3^x^	128.3 (3)	Sn3^xiv^—Sn2—Bi1^xi^	90.000 (7)
Sn3^ix^—Bi1—Sn3^x^	98.2 (6)	Sn3—Sn2—Bi1^xi^	90.000 (7)
Sn3^vi^—Bi1—Sn3^x^	128.3 (3)	Sn1^ix^—Sn2—Bi1^xi^	120.0
Sn3—Bi1—Sn3^xiii^	119.2 (5)	Pb1^ix^—Sn2—Bi1^xi^	120.0
Sn3^i^—Bi1—Sn3^xiii^	60.8 (5)	Bi1^ix^—Sn2—Bi1^xi^	120.0
Sn2^ix^—Bi1—Sn3^xiii^	115.87 (13)	Sn1^x^—Sn2—Bi1^xi^	120.0
Sn2^x^—Bi1—Sn3^xiii^	29.2 (5)	Sn1^xi^—Sn2—Bi1^xi^	0.0
Sn2^xi^—Bi1—Sn3^xiii^	115.87 (13)	Pb1^x^—Sn2—Bi1^xi^	120.0
Sn2—Bi1—Sn3^xiii^	119.2 (5)	Pb1^xi^—Sn2—Bi1^xi^	0.0
Sn2^xii^—Bi1—Sn3^xiii^	60.8 (5)	Bi1^x^—Sn2—Bi1^xi^	120.0
Sn3^iv^—Bi1—Sn3^xiii^	98.2 (6)	Sn3^xiv^—Sn2—Bi1	180.0
Sn3^ix^—Bi1—Sn3^xiii^	128.3 (3)	Sn3—Sn2—Bi1	0.000 (6)
Sn3^vi^—Bi1—Sn3^xiii^	98.2 (6)	Sn1^ix^—Sn2—Bi1	90.0
Sn3^x^—Bi1—Sn3^xiii^	58.5 (10)	Pb1^ix^—Sn2—Bi1	90.0
Sn3—Pb1—Sn3^i^	180.0	Bi1^ix^—Sn2—Bi1	90.0
Sn3—Pb1—Sn2^ix^	90.000 (1)	Sn1^x^—Sn2—Bi1	90.0
Sn3^i^—Pb1—Sn2^ix^	89.999 (1)	Sn1^xi^—Sn2—Bi1	90.0
Sn3—Pb1—Sn2^x^	90.000 (4)	Pb1^x^—Sn2—Bi1	90.0
Sn3^i^—Pb1—Sn2^x^	90.000 (4)	Pb1^xi^—Sn2—Bi1	90.0
Sn2^ix^—Pb1—Sn2^x^	120.0	Bi1^x^—Sn2—Bi1	90.0
Sn3—Pb1—Sn2^xi^	90.000 (5)	Bi1^xi^—Sn2—Bi1	90.0
Sn3^i^—Pb1—Sn2^xi^	90.000 (4)	Sn2—Sn3—Bi1	180.0
Sn2^ix^—Pb1—Sn2^xi^	120.0	Sn2—Sn3—Pb1	180.0
Sn2^x^—Pb1—Sn2^xi^	120.0	Bi1—Sn3—Pb1	0.0
Sn3—Pb1—Sn2	0.000 (6)	Sn2—Sn3—Sn1	180.0
Sn3^i^—Pb1—Sn2	180.0	Bi1—Sn3—Sn1	0.0
Sn2^ix^—Pb1—Sn2	90.0	Pb1—Sn3—Sn1	0.0
Sn2^x^—Pb1—Sn2	90.0	Sn2—Sn3—Sn3^ix^	90.2 (13)
Sn2^xi^—Pb1—Sn2	90.0	Bi1—Sn3—Sn3^ix^	89.8 (13)
Sn3—Pb1—Sn2^xii^	180.0	Pb1—Sn3—Sn3^ix^	89.8 (13)
Sn3^i^—Pb1—Sn2^xii^	0.000 (1)	Sn1—Sn3—Sn3^ix^	89.8 (13)
Sn2^ix^—Pb1—Sn2^xii^	90.0	Sn2—Sn3—Pt1^xv^	90.1 (6)
Sn2^x^—Pb1—Sn2^xii^	90.0	Bi1—Sn3—Pt1^xv^	89.9 (6)
Sn2^xi^—Pb1—Sn2^xii^	90.0	Pb1—Sn3—Pt1^xv^	89.9 (6)
Sn2—Pb1—Sn2^xii^	180.0	Sn1—Sn3—Pt1^xv^	89.9 (6)
Sn3—Pb1—Sn3^iv^	119.2 (5)	Sn3^ix^—Sn3—Pt1^xv^	179.7 (19)
Sn3^i^—Pb1—Sn3^iv^	60.8 (5)	Sn2—Sn3—Pt1^xvi^	90.1 (6)
Sn2^ix^—Pb1—Sn3^iv^	29.2 (5)	Bi1—Sn3—Pt1^xvi^	89.9 (6)
Sn2^x^—Pb1—Sn3^iv^	115.87 (13)	Pb1—Sn3—Pt1^xvi^	89.9 (6)
Sn2^xi^—Pb1—Sn3^iv^	115.87 (13)	Sn1—Sn3—Pt1^xvi^	89.9 (6)
Sn2—Pb1—Sn3^iv^	119.2 (5)	Sn3^ix^—Sn3—Pt1^xvi^	60.000 (1)
Sn2^xii^—Pb1—Sn3^iv^	60.8 (5)	Pt1^xv^—Sn3—Pt1^xvi^	120.000 (4)
Sn3—Pb1—Sn3^ix^	60.8 (5)	Sn2—Sn3—Pt1^vii^	90.1 (6)
Sn3^i^—Pb1—Sn3^ix^	119.2 (5)	Bi1—Sn3—Pt1^vii^	89.9 (6)
Sn2^ix^—Pb1—Sn3^ix^	29.2 (5)	Pb1—Sn3—Pt1^vii^	89.9 (6)
Sn2^x^—Pb1—Sn3^ix^	115.87 (13)	Sn1—Sn3—Pt1^vii^	89.9 (6)
Sn2^xi^—Pb1—Sn3^ix^	115.87 (13)	Sn3^ix^—Sn3—Pt1^vii^	60.000 (5)
Sn2—Pb1—Sn3^ix^	60.8 (5)	Pt1^xv^—Sn3—Pt1^vii^	120.000 (4)
Sn2^xii^—Pb1—Sn3^ix^	119.2 (5)	Pt1^xvi^—Sn3—Pt1^vii^	120.000 (4)
Sn3^iv^—Pb1—Sn3^ix^	58.5 (10)	Sn2—Sn3—Sn3^x^	90.2 (13)
Sn3—Pb1—Sn3^vi^	119.2 (5)	Bi1—Sn3—Sn3^x^	89.8 (13)
Sn3^i^—Pb1—Sn3^vi^	60.8 (5)	Pb1—Sn3—Sn3^x^	89.8 (13)
Sn2^ix^—Pb1—Sn3^vi^	115.87 (13)	Sn1—Sn3—Sn3^x^	89.8 (13)
Sn2^x^—Pb1—Sn3^vi^	115.87 (13)	Pt1^xv^—Sn3—Sn3^x^	60.0
Sn2^xi^—Pb1—Sn3^vi^	29.2 (5)	Pt1^xvi^—Sn3—Sn3^x^	60.000 (1)
Sn2—Pb1—Sn3^vi^	119.2 (5)	Pt1^vii^—Sn3—Sn3^x^	179.7 (19)
Sn2^xii^—Pb1—Sn3^vi^	60.8 (5)	Sn2—Sn3—Sn3^xi^	90.2 (13)
Sn3^iv^—Pb1—Sn3^vi^	98.2 (6)	Bi1—Sn3—Sn3^xi^	89.8 (13)
Sn3^ix^—Pb1—Sn3^vi^	128.3 (3)	Pb1—Sn3—Sn3^xi^	89.8 (13)
Sn3—Pb1—Sn3^x^	60.8 (5)	Sn1—Sn3—Sn3^xi^	89.8 (13)
Sn3^i^—Pb1—Sn3^x^	119.2 (5)	Pt1^xv^—Sn3—Sn3^xi^	59.999 (4)
Sn2^ix^—Pb1—Sn3^x^	115.87 (13)	Pt1^xvi^—Sn3—Sn3^xi^	179.7 (19)
Sn2^x^—Pb1—Sn3^x^	29.2 (5)	Pt1^vii^—Sn3—Sn3^xi^	60.000 (6)
Sn2^xi^—Pb1—Sn3^x^	115.87 (13)	Sn2—Sn3—Sn3^xiv^	0.0
Sn2—Pb1—Sn3^x^	60.8 (5)	Bi1—Sn3—Sn3^xiv^	180.0
Sn2^xii^—Pb1—Sn3^x^	119.2 (5)	Pb1—Sn3—Sn3^xiv^	180.0
Sn3^iv^—Pb1—Sn3^x^	128.3 (3)	Sn1—Sn3—Sn3^xiv^	180.0
Sn3^ix^—Pb1—Sn3^x^	98.2 (6)	Sn3^ix^—Sn3—Sn3^xiv^	90.2 (13)
Sn3^vi^—Pb1—Sn3^x^	128.3 (3)	Pt1^xv^—Sn3—Sn3^xiv^	90.1 (6)
Sn3—Pb1—Sn3^xiii^	119.2 (5)	Pt1^xvi^—Sn3—Sn3^xiv^	90.1 (6)
Sn3^i^—Pb1—Sn3^xiii^	60.8 (5)	Pt1^vii^—Sn3—Sn3^xiv^	90.1 (6)
Sn2^ix^—Pb1—Sn3^xiii^	115.87 (13)	Sn3^x^—Sn3—Sn3^xiv^	90.2 (13)
Sn2^x^—Pb1—Sn3^xiii^	29.2 (5)	Sn3^xi^—Sn3—Sn3^xiv^	90.2 (13)
Sn2^xi^—Pb1—Sn3^xiii^	115.87 (13)	Sn2—Sn3—Sn3^i^	180.0
Sn2—Pb1—Sn3^xiii^	119.2 (5)	Bi1—Sn3—Sn3^i^	0.0
Sn2^xii^—Pb1—Sn3^xiii^	60.8 (5)	Pb1—Sn3—Sn3^i^	0.0
Sn3^iv^—Pb1—Sn3^xiii^	98.2 (6)	Sn1—Sn3—Sn3^i^	0.0
Sn3^ix^—Pb1—Sn3^xiii^	128.3 (3)	Sn3^ix^—Sn3—Sn3^i^	89.8 (13)
Sn3^vi^—Pb1—Sn3^xiii^	98.2 (6)	Pt1^xv^—Sn3—Sn3^i^	89.9 (6)
Sn3^x^—Pb1—Sn3^xiii^	58.5 (10)	Pt1^xvi^—Sn3—Sn3^i^	89.9 (6)
Sn3—Sn1—Sn3^i^	180.0	Pt1^vii^—Sn3—Sn3^i^	89.9 (6)
Sn3—Sn1—Sn2^ix^	90.000 (1)	Sn3^x^—Sn3—Sn3^i^	89.8 (13)
Sn3^i^—Sn1—Sn2^ix^	89.999 (1)	Sn3^xi^—Sn3—Sn3^i^	89.8 (13)
Sn3—Sn1—Sn2^x^	90.000 (4)	Sn3^xiv^—Sn3—Sn3^i^	180.0

Symmetry codes: (i) *x*, *y*, −*z*+1/2; (ii) −*x*, −*y*, *z*−1/2; (iii) *x*−1, *y*−1, −*z*+1/2; (iv) −*x*+1, −*y*+1, *z*−1/2; (v) *x*, *y*−1, −*z*+1/2; (vi) −*x*, −*y*+1, *z*−1/2; (vii) −*x*, −*y*, *z*+1/2; (viii) −*x*, −*y*, −*z*; (ix) −*x*+1, −*y*+1, −*z*+1; (x) −*x*+1, −*y*+2, −*z*+1; (xi) −*x*, −*y*+1, −*z*+1; (xii) *x*, *y*, *z*−1; (xiii) −*x*+1, −*y*+2, *z*−1/2; (xiv) *x*, *y*, −*z*+3/2; (xv) −*x*, −*y*+1, *z*+1/2; (xvi) −*x*+1, −*y*+1, *z*+1/2.
